# Melting of PCMs Embedded in Copper Foams: An Experimental Study

**DOI:** 10.3390/ma14051195

**Published:** 2021-03-04

**Authors:** Andrea Diani, Luisa Rossetto

**Affiliations:** Department of Industrial Engineering, University of Padova, via Venezia 1, 35131 Padova, Italy; andrea.diani@unipd.it

**Keywords:** phase change material, copper foam, paraffin, electronics cooling

## Abstract

A smart possible way to cool electronics equipment is represented by passive methods, which do not require an additional power input, such as Phase Change Materials (PCMs). PCMs have the benefit of their latent heat being exploited during the phase change from solid to liquid state. This paper experimentally investigates the melting of different PCMs having different melting temperatures (42, 55 and 64 °C). Two copper foams, having 10 PPI and relative densities of 6.7% and 9.5%, i.e., porosities of 93.3% and 90.5%, respectively, are used to enhance the thermal conductivity of PCMs. The block composed by the PCM and the copper foam is heated from one side, applying three different heat fluxes (10, 15 and 20 kW m^−2^): the higher the heat flux, the higher the temperature reached by the heated side and the shorter the time for a complete melting of the PCM. The copper foam with a relative density of 9.5% shows slightly better performance, whereas the choice of the melting temperature of the PCM depends on the time during which the passive cooling system must work. The effect of the foam material is also presented: a copper foam presents better thermal performances than an aluminum foam with the same morphological characteristics. Finally, experimental dimensionless results are compared against values predicted by a correlation previously developed.

## 1. Introduction

The use of Phase Change Materials (PCMs) for electronics cooling applications is an appealing topic nowadays. One of its possible implementations is in the fanless configurations that the electronics industry is now pushing into the market. The heat transfer associated with the phase change from solid to liquid state is much higher than the sensible enthalpy change that can be exploited in the case of heat sinks based on natural/forced convection processes. However, the majority of PCMs are characterized by low thermal conductivities, with consequent high temperatures associated with the heated side. Therefore, solid media inserted into the PCM should be used to enhance the conductivity of the PCM block.

Among the possible solid media, open-cell metal foams are one of the possible solutions. Metal foams are cellular materials, with cells generated by ligaments which are randomly oriented and distributed. Two main parameters classify open-cell foams: PPI and relative density. PPI is the number of pores that can be counted in a linear inch. The relative density is the ratio between the density of the foam and the density of the material which the foam is made of. Generally speaking, typical values of relative density vary from 2% to 15%. Higher values of relative density may lead to closed cells. The values of the relative density also affect the shape of the section of the ligaments [1]. The present research is focused on Duocel^®^ copper foams, for which the typical values of PPI range between 5 and 40, whereas the relative density varies from 3% to 12% [2].

Most of the works related to the phase change process solid-liquid/liquid-solid inside metal foams are experimental and/or numerical studies. Till now, just a few studies tried to analytically/empirically model the phase change process, even because these models are typical for the specific set up. As stated by Zhao [3], high-porosity open-cell metal foams are considered as one of the most promising materials to enhance the heat transfer with PCM due to their high thermal conductivities and high surface area densities.

Zhou and Zhao [4] experimentally studied the heat transfer characteristics of a paraffin and of a hydrated salt embedded in open-cell metal foams and expanded graphite. The experimental results indicated that, compared to the case without any insert, adding a porous material can enhance the heat transfer rate of the PCM: at the same heat flux, the temperature of the heater is lower, and the melting time is shortened.

Li et al. [5] experimentally and numerically investigated the melting phase change heat transfer of paraffin embedded inside copper foams with porosities higher than 0.90. It was shown that the melting heat transfer is enhanced by the higher thermal conductivity of the metallic foams, even though their presence inhibits the natural convection. The numerical model was able to predict the experimental findings.

Another experimental and numerical study was proposed by Chen et al. [6], who studied the melting process at the pore scale using an infrared camera to monitor the temperature field and an optical microscope to observe the melting evolution of the PCM. The good thermal performance of the system was mainly due to the augmented thermal conductivity of the PCM block due to the presence of the solid matrix. Other experimental and numerical studies about PCM melting inside metal foams can be found in Hu and Patnaik [7], Yang and Garimella [8], Sundarram and Li [9] and Mancin et al. [10].

Considering more recent papers which analyze the heat transfer of PCMs embedded in metal foams, Zhu et al. [11] investigated the transient performance of a heat sink filled with a copper foam and a PCM. They considered two copper foams (15 PPI and 30 PPI) with a porosity of about 96% and a PCM with a melting temperature of 46 °C. The effect of the filling ratio both on the heating process and on the cool-down process was investigated. The experimental results revealed that the porosity had an insignificant effect at lower heating powers, whereas a better thermal performance can be achieved with larger pore sizes at high heating powers, whereas no effect of PPI was observed during cool-down. A partial filling strategy can be considered to reduce costs while maintaining a good thermal performance. The effect of the filling ratio, as well as of the porosity of the foam, was numerically studied by Joshi and Rathod [12]. Numerical results showed that only the lowest filling ratio tested (0.25 of the height) led to a benefit on the thermal performance. Further increases of the filling ratio from 0.75 to 1 times the height of the foam required about the same melting time. As a general statement, the total melting rate was found to decrease as both filling ratio and porosity decreased.

Yang et al. [13] considered the heat transfer in solidification of PCMs embedded in metal foams with the insertion of pin fins. The experimental results indicated that the insertion of pin fins greatly improved the solidification process regardless of gradient in pore parameters. The solidification rate could be further improved acting on the gradient in porosity rather than of on the parent material. The best structure was recommended to be a pin fins-metal foam hybrid matrix with gradient in metal foam porosity.

Marri and Balaji [14] experimentally and numerically studied the thermal performance of a PCM-metal foam heat sink with a cylindrical shape. Studies were conducted for aluminum foams with different PPI (8, 14 and 20), different porosities (90%, 94% and 97%), encapsulated with n-eicosane as the phase change material. The results indicated that either decreasing the porosity or increasing the PPI from the bottom to the top enhanced the thermal performance of the heat sink compared to the case with uniform porosity and PPI.

Iasiello et al. [15] presented experimental and numerical results on PCMs embedded in aluminum foams under different heat fluxes, porosities, PPIs and orientation. An infrared camera was used to capture the temperature distribution with the aim of tracking the melting front.

Examples of practical applications of PCMs can be found in Madruga [16], who demonstrated how a PCM can improve the performance of a Thermoelectric Generator joined to a thermal storage unit; in Carmona et al. [17], who considered a latent heat thermal energy storage with phase change materials incorporated in a domestic hot water systems; and in Dardir et al. [18], who developed a new concept of PCM-to-air heat exchanger aimed at increasing the cooling charging power of the system.

Just a few works tried to empirically or analytically model the phase change process from solid to liquid state with solid media inserted into the PCM. Among these works, Mallow et al. [19], based on their experimental data collected during melting of two waxes with melting temperature of 37 and 54 °C inside alumina and graphite foams, proposed an empirical correlation to correlate a dimensionless temperature to a modified Fourier number. A similar approach was implemented by Diani and Campanale [20]. Based on experimental data during melting of three paraffins having three different melting temperatures, embedded in aluminum foams with different PPI and approximately the same porosity, they proposed a correlation to correlate a dimensionless temperature to the product between the Stefan number and the Fourier number.

This paper presents experimental results during the melting process of three paraffin waxes, namely RT42, RT55 and RT64HC, into two copper foams with the same linear porosity (10 PPI), but different volumetric porosity (93.3% and 90.5%), in order to catch the effect of this geometrical parameter. The comparison against the data obtained with aluminum foams shows also the effect of the foam material. The experimental results will permit us to validate empirical correlations which also take into account the volumetric porosity and foam material.

## 2. Copper Foams and Paraffin Waxes

**Figure 1:**
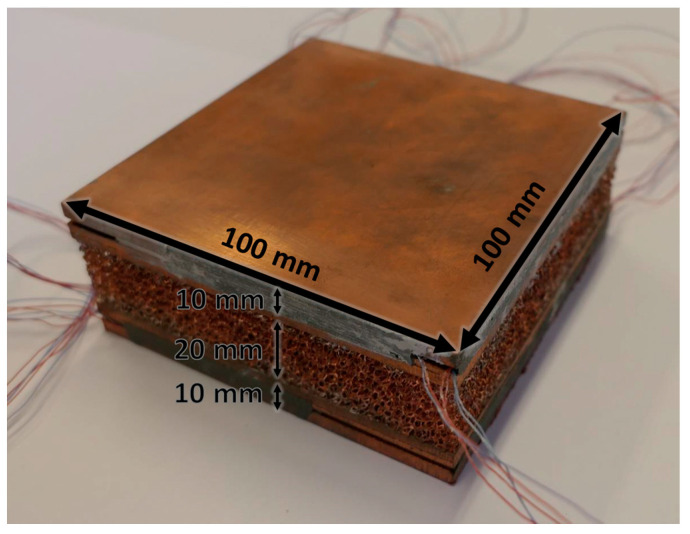
Copper foam with geometrical dimensions.

Two Duocel^®^ copper (C10100 alloy) foams were tested during melting of phase change materials. The tested foams are made in a sandwichlike arrangement, i.e., the copper foam is brazed between two copper plates: the core of each foam has a height of 20 mm, whereas each copper plate has a height of 10 mm. The copper foams, and consequently the copper plates, have a square base with an edge of 100 mm. A picture of one of the two tested copper foams in the sandwichlike arrangement is reported in Figure 1, as well as its geometrical sizes.

Holes were drilled inside the plates, to host T-type calibrated thermocouples to monitor the wall temperature distribution during the heating process. Further details about the arrangement of the thermocouples are reported in [20].

The two copper foams have the same number of pores per linear inch (10), but different relative density *ρ_r_* (6.7% and 9.5%), thus allowing us to better understand the effect of this parameter on the melting behavior of PCMs embedded in metal foams. The main geometrical characteristics of the tested copper foams are listed in Table 1, where the volumetric porosity *ε* is the ratio between the volume occupied by the empty spaces and the total volume (foam and empty spaces). The two foams are named Cu-10-6.7 and Cu-10-9.5, where the first term indicates the parent material, the second one indicates the number of pores per linear inch, and the third one is the relative density in percentage.

The described copper foams are the solid media used to enhance the thermal conductivity of three different paraffine waxes, which are used as phase change material. The paraffin waxes used in the present research are named RT42, RT55 and RT64HC. The number that appears in the name means the characteristic melting temperature of the paraffin, even if it would be better to talk about a melting temperature range instead of a single melting temperature. These PCMs are chemically inert with a stable performance through the phase change cycles. Table 2 lists the main thermophysical characteristics of the tested PCMs.

## 3. Experimental Set Up

The experimental set up was designed to carry out transient experimental tests during heating of the module while recording the temperatures of both the heated side and of the paraffin melting inside the module.

To limit the heat losses through the ambient surroundings as much as possible, a Teflon case was developed to host both the paraffin embedded in the metal foam and the heating element. A schematic of the Teflon module can be found in Diani and Campanale [20].

Three different heat fluxes (10, 15 and 20 kW m^−2^) were supplied to the foam block by means of an electrical heater. It consists of a copper plate inside which a guide was milled to host a nickel-chrome wire resistance. A schematic of the electrical heater can be found in Diani and Campanale [20]. This electrical resistance is electrically insulated from the plate with a heat shrink sheath and inserted into the guide with thermal grease. A thin copper plate is screwed to the plate to enclose the electrical resistance. Therefore, samples are electrically heated by the Joule effect. The electrical heater is connected to a DC (Direct Current) power supplier. The supplied electrical power is measured by two distinct measurements of Electric Differential Potential (EDP). The first one is across the nickel-chrome wire inserted into the heater, and it permits to know the voltage *V*. The second one is across a calibrated reference resistance (shunt), which is in series with the electric heater: this EDP measurement allows us to calculate the current *I* flowing into the circuit from the Ohm’s law, since the shunt has a known reference resistance. Consequently, the supplied electric power can be calculated as the product between the voltage *V* and the current *I*. Heat fluxes are calculated by dividing the electric power by the area of the heated plate, i.e., 100 × 100 mm^2^.

**Figure 2:**
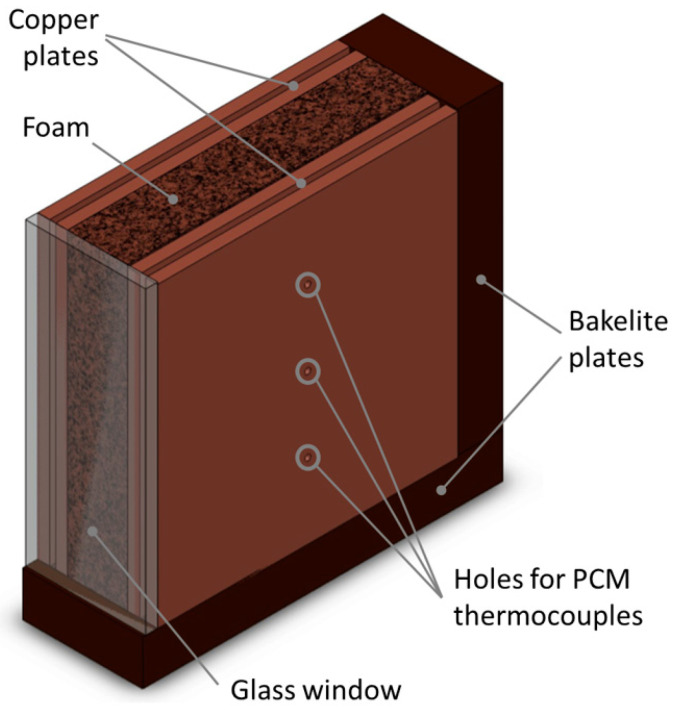
Foam block with bakelite plates.

The metal foams are tested in an upright position, as reported in Figure 2, and so they are laterally heated (from the left side of the figure). In order to enclose the sandwichlike arrangement of the foam block, two bakelite plates are glued to the foam block (one plate on the bottom, and the other one on the rear side) as reported in Figure 2. A glass window is glued on the front side, and it permits us to visualize the phase change process occurring inside the foam. The top part is left open to permit the filling of the foam with the paraffin wax. Samples are considered filled when the liquid level reaches the top part of the foam.

Besides the thermocouples inserted into the copper plates to monitor the wall temperature, three additional T-type thermocouples were inserted into as many holes drilled into the right side of the PCM/foam block to monitor the temperature distribution of the phase change material. These thermocouples were inserted on the right side, and the correspondent three holes in the right copper plate represent their location (see Figure 2). These thermocouples (accuracy of ± 0.5 K) are sheathed in stainless steel to give stiffness to reach the middle section of the PCM/foam block. All the implemented thermocouples are connected to a Kaye 170 ice point reference. All the signals, i.e., of the thermocouples and of the two EDP measurements, are recorded using a HP34970A multimeter.

## 4. Experimental Results

Every foam structure is tested with three different heat fluxes (10, 15 and 20 kW m^−2^), each one with three different paraffin waxes having different melting temperatures (42, 55 and 64 °C). Two copper foams are tested (Cu-10-6.7 and Cu-10-9.5). Therefore, a total amount of 18 experimental tests are carried out, allowing us to understand the effect of heat flux, melting temperature and foam porosity on the melting behavior. Furthermore, a comparison against an aluminum foam with 10 PPI and a relative density of 7.4% is presented, allowing us to understand the effect of the foam material.

### 4.1. Experimental Procedure

The first step is the filling of the foam structure with the paraffin, and this procedure is deemed concluded once the liquid paraffin wax fills the entire foam structure. Once filled, the module is left to cool down until ambient temperature, i.e., the heating process starts from ambient temperature. The data acquisition system starts, as well as the recording process starts, as soon as the DC current generator is switched on. The data acquisition system permits us to record every signal approximately every 4 s. Each experimental test is deemed concluded as soon as all the PCM is melted inside the foam. Besides the recorded signals, pictures are taken in order to monitor the melting process during the heating process through the glass window. These pictures permit us to observe how the melting front propagates inside the PCM during the heating process. The following experimental results will be given in terms of temperature profiles (average value of the temperature of the heated side or temperatures of the PCM) during the heating process until the PCM inside the structures is fully melted.

### 4.2. Melting Behavior

**Figure 3:**
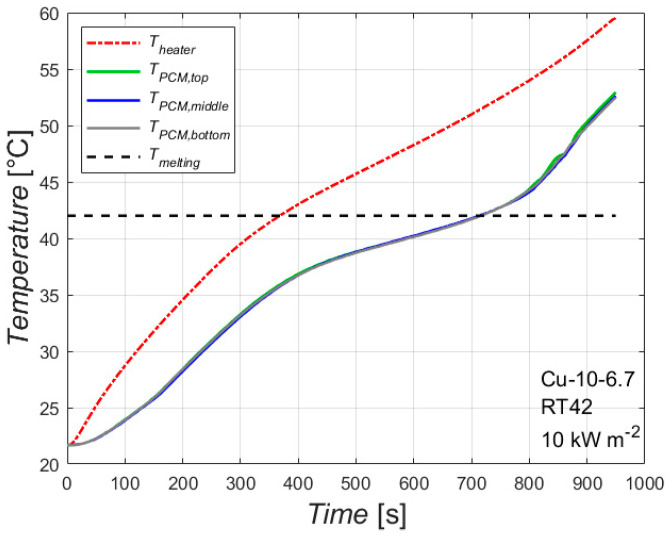
Temperature profiles during melting of the paraffin RT42 inside the 10 PPI copper foam with a porosity of 0.933 with an imposed heat flux of 10 kW m^−2^.

Figure 3 reports the temperatures of the heated side and of the PCM at three different locations plotted against the time for the foam Cu-10-6.7 with the paraffin having a melting temperature of 42 °C with an imposed heat flux of 10 kW m^−2^, i.e., 100 W. The analysis of the temperature trends that will be explained in this paragraph can be extended for the other heat fluxes and paraffins as well as for the other foam. The time needed to completely melt the PCM and the temperature reached by the heated side will depend on the combination of foam, paraffin and heat flux. The effect of these parameters will be explained in the next paragraphs.

The red line represents the average temperature of the plate in contact with the heater. It is worth underlining that all the recorded wall temperatures are within ± 1 K, which means that the heating is constant along the height of the sample during the heating process, allowing us to consider an average temperature for the heated side instead of single wall temperatures. Since the experimental tests start at ambient temperature, the temperature recorded at time *t* = 0 s represents the ambient temperature. The temperature of the heated side increases as the heating process proceeds, and its slope changes when the temperature reaches the temperature range of the melting process. The first part of the test, until the melting temperature is reached, is characterized by only sensible heat. After the melting temperature, latent heat also starts to be involved, with a consequent change of the slope of the curve of the heated side temperature.

The melting temperature of this paraffin is between 38 °C and 43 °C: this is reflected on the three lines (green, blue and grey) related to the temperatures of the PCM recorded by the thermocouples which measure the PCM temperature in the middle of the PCM/foam block at three different heights: 25, 50 and 75 mm. As can be seen in the figure, there are two changes of the slope of these lines: the first one is in correspondence with the lower value of the melting temperature range (when the melting process starts in that location), whereas the second one is in correspondence with the upper value of the melting temperature range (when the melting process ends in that location). Furthermore, it can be noted that these three lines collapse into one line during the heating process: this means that the melting front propagates as a vertical line at least until the centerline of the PCM/foam block is reached.

**Figure 4:**
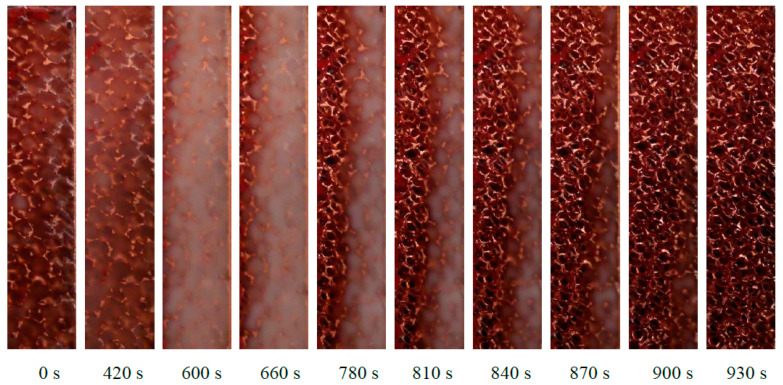
Melting front of the paraffin RT42 inside the 10 PPI copper foam with a porosity of 0.933 with an imposed heat flux of 10 kW m^−2^.

Figure 3 can be coupled with Figure 4, which shows some pictures taken from the glass window of the module at different moments. When the melting of the PCM starts, there is a change of the transparency of the paraffin: at 420 s for instance, the temperature of the heated side is higher than the melting temperature, and therefore the paraffin on the left side has a different transparency compared to the paraffin on the right side. The sequence of these pictures demonstrates that, for the considered physical sizes of the foam block, the melting front is quite parallel to the heated side, meaning that the 20 mm thick foam structure tends to inhibit the natural convection of the PCM. As time goes by, the melted liquid paraffin tends to overcome the solid paraffin due to volume variation from solid to liquid conditions, and this may explain the reason why the last part of PCM to melt is on the bottom right corner of the foam.

Similar conclusions can be drawn for the other foam and for the other combinations of heat fluxes and melting temperatures. The time needed to completely melt the PCM and the temperatures at the end of the heating process will change depending on the test conditions. Table 3 reports, for each combination of foam, paraffin and heat flux, the temperatures of the heated side at the beginning and at the end of the test, as well as the time needed to completely melt the phase change material inside the foam. Generally speaking, the foam Cu-10-9.5 requires slightly shorter times to completely melt the paraffin, except for the case with the paraffin RT42 with an imposed heat flux of 10 kW m^−2^, since in this case the initial temperature for the test with the foam Cu-10-9.5 is about 2.3 K lower than that of the test with the foam Cu-10-6.7. The effect of each parameter will be discussed in the next paragraphs.

### 4.3. Effect of Heat Flux

**Figure 5:**
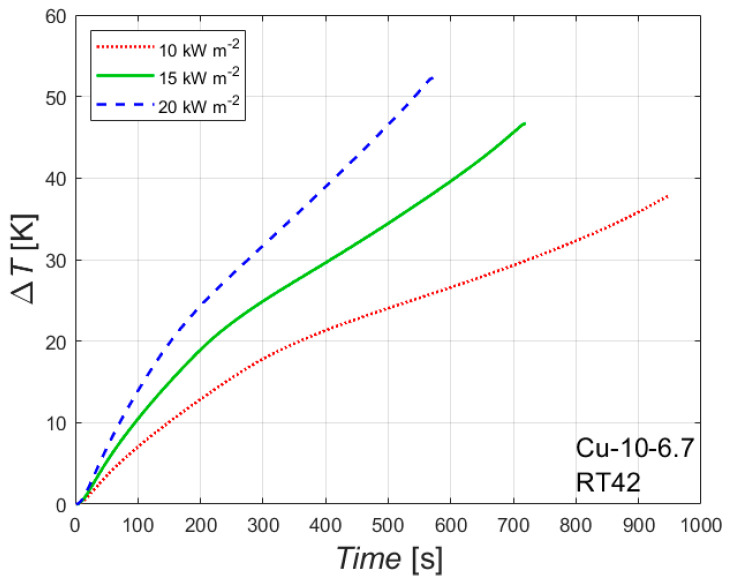
Effect of heat flux on the difference between wall and initial temperatures for the 10 PPI copper foam with a porosity of 0.933 with the paraffin RT42.

The effect of the heat flux on the melting process is reported in Figure 5, which reports Δ*T* versus time for the foam Cu-10-6.7 embedded with the paraffin RT42. Δ*T* represents the difference between the temperature of the wall in contact with the heater and the initial temperature. Considering Δ*T* instead of the temperature itself allows us to compare data with different initial temperatures. However, the three experimental conditions considered in Figure 5 have initial temperatures within ± 2.1 K.

As expected, the heat flux affects both the time needed to completely melt the PCM inside foam and the temperature reached by the heated side at the end of the melting process: the higher the heat flux, the shorter the time needed for a complete melting and the higher the temperature of the heated wall. Similar conclusions, but with different values of final temperatures and times needed for a complete melting of the PCM, can be drawn from Table 3 for the other foam and for the other paraffin waxes.

### 4.4. Effect of Melting Temperature

**Figure 6:**
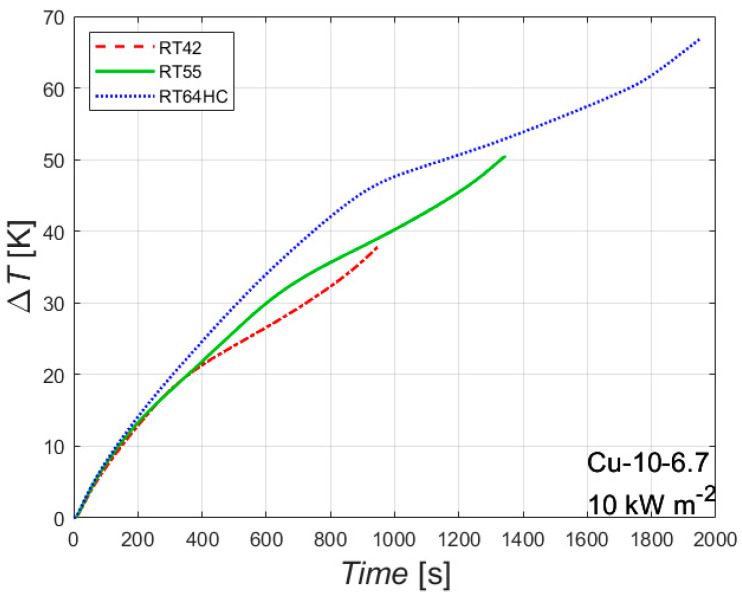
Effect of melting temperature on the difference between wall and initial temperatures for the 10 PPI copper foam with a porosity of 0.933 with an imposed heat flux of 10 kW m^−2^.

The effect of the melting temperature is shown in Figure 6. The figure reports the difference between the wall temperature of the heated side and the initial temperature plotted against the time, for the foam Cu-10-6.7 with an imposed heat flux of 10 kW m^−2^ for three different PCMs having different melting temperatures. The melting temperature has almost no effect on the first part of the experimental test, where only sensible heat is involved, since all the three tested paraffins have similar thermophysical properties for the solid state. As soon as the melting temperature is reached, the slope of the curve changes, and, as a result, the curve related to the paraffin RT42 is the first one that changes its slope, followed by RT55 and RT64HC.

The melting temperature affects both the time needed to completely melt the PCM and the temperature of the heated side at the end of the melting process. The lower the melting temperature, the sooner the melting process ends, since a lower amount of sensible heat is needed to start the melting process. The higher the melting temperature, the higher the temperature of the heated side at the end of the melting process. These considerations should be considered in order to optimize the choice of the melting temperature of a PCM in real applications. Considering, for instance, the working conditions reported in Figure 6, the paraffin RT42 can be considered the best choice until 950 s, the paraffin RT55 from 950 s to approximately 1400 s, the paraffin RT64HC for longer times, i.e., the choice of the melting temperature of the PCM depends on the time during which the metal foam/PCM based heat sink should work. Similar trends can be drawn for the other foams and for the other heat fluxes.

### 4.5. Effect of Relative Density

**Figure 7:**
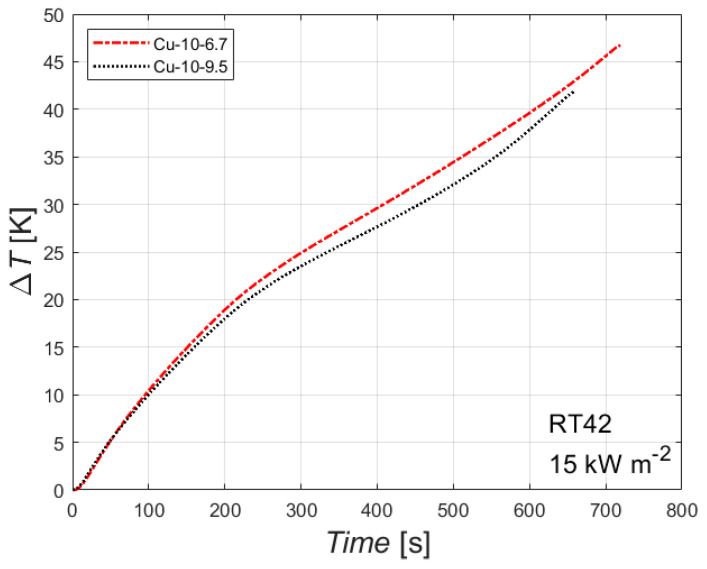
Effect of foam relative density, i.e., of porosity, on the difference between wall and initial temperatures for the paraffin RT42 with an imposed heat flux of 15 kW m^−2^ for the two 10 PPI copper foams.

The effect of the foam’s relative density is reported in Figure 7. The figure reports the difference between the temperature of the heated side and the initial temperature plotted against the time for the paraffin RT42 with an imposed heat flux of 15 kW m^−2^ for two copper foams with the same number of PPI but different relative density.

The difference of the foam relative densities for the two tested samples is quite limited (6.7% and 9.5%), and so there is a small difference between the two curves. However, the effect of the foam’s relative density seems to be clear: the higher the foam relative density, the better the thermal performance, i.e., the lower the difference between the heated side and the initial temperature. This can be attributed to the higher thermal conductivity of the sample with the highest foam relative density, which leads to lower temperatures of the heated side at constant heat flux and PCM. The higher the relative densities, the shorter the time needed to complete the melting of the PCM embedded in the foam matrix. Larger differences in foams’ relative densities may have led to larger differences in the thermal performances. Similar conclusions can be drawn for the other combinations of phase change material and heat flux.

### 4.6. Effect of Foam Material 

**Figure 8:**
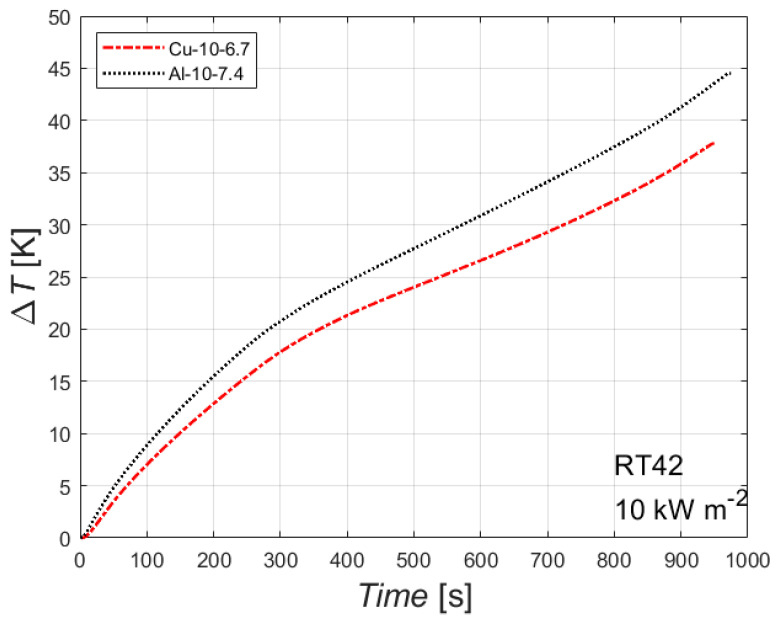
Effect of foam parent material for two 10 PPI foams (copper foam with porosity of 0.933, aluminum foam with porosity of 0.926) on the difference between wall and initial temperatures for the paraffin RT42 with an imposed heat flux of 10 kW m^−2^.

The effect of the foam parent material is shown in Figure 8. The figure reports the difference between the heated wall temperature and the initial temperature, plotted against the time, for the paraffin with a melting temperature of 42 °C with an imposed heat flux of 10 kW m^−2^, for two foams with different parent material but with the same number of PPI and about the same relative density (6.7% for the copper foam and 7.4% for the aluminum foam). The data for the aluminum foam are borrowed from Diani and Campanale [20]. The parent material has no effect on the time needed to completely melt the PCM inside the foam block, but it affects the temperature difference: the copper foam shows a lower temperature difference, i.e., a lower temperature on the heated side, which is more favorable for a real application. This can be attributed to the higher thermal conductivity of the parent material, which enhances the thermal performance at constant heat flux and melting temperature. Similar conclusions can be drawn for the other combinations of heat flux and melting temperature.

## 5. Empirical Modeling

In this section, the experimental results of time needed for a complete melting of the PCM and the temperature reached by the heated side at the end of the heating process are compared against the values predicted by the correlation proposed by Diani and Campanale [20]. The model correlates a dimensionless temperature, *θ*, to the product between Fourier number, Fo, and Stefan number, Ste, as, Equation (1):

(1) $$\theta =1.9073\cdot {\left(\mathrm{Fo}\cdot \mathrm{Ste}\right)}^{-0.717}$$

where the dimensionless numbers can be expressed as, Equations (2) and (3):

(2) $$\theta =\frac{T_{f}-T_{melt}}{T_{melt}-T_{i}}$$

(3) $$\mathrm{Fo}\cdot \mathrm{Ste}=\frac{k_{efff}\cdot t_{melt}\cdot \left(T_{melt}-T_{i}\right)}{\rho_{eff}\cdot h^{2}\cdot L_{eff}}$$

with *T_f_* final temperature of the heated side, *T_melt_* melting temperature of the PCM, *T_i_* initial temperature, *k_eff_* effective thermal conductivity, which considers both the contribution of the foam parent material and of the PCM, *t_melt_* time needed to completely melt the PCM, *ρ_eff_* effective density, which considers both parent material and PCM, *h* foam thickness (20 mm in the actual case), *L_eff_* effective latent heat. Further details can be found in Diani and Campanale [20]. The effective thermal conductivity *k_eff_* is calculated as suggested by the manufacturer [2], and as reported by Mallow et al. [19], as, Equation (4):

(4) $$k_{eff}=0.33\cdot k_{solid}\cdot \left(1-\varepsilon \right)$$

where *k_solid_* is the thermal conductivity of the parent material. In the present case, considering a copper thermal conductivity of 390 W m^−1^ K^−1^, the effective thermal conductivity of the foam Cu-10-6.7 is 8.6 W m^−1^ K^−1^, whereas it is 12.2 W m^−1^ K^−1^ for the foam Cu-10-9.5.

**Figure 9:**
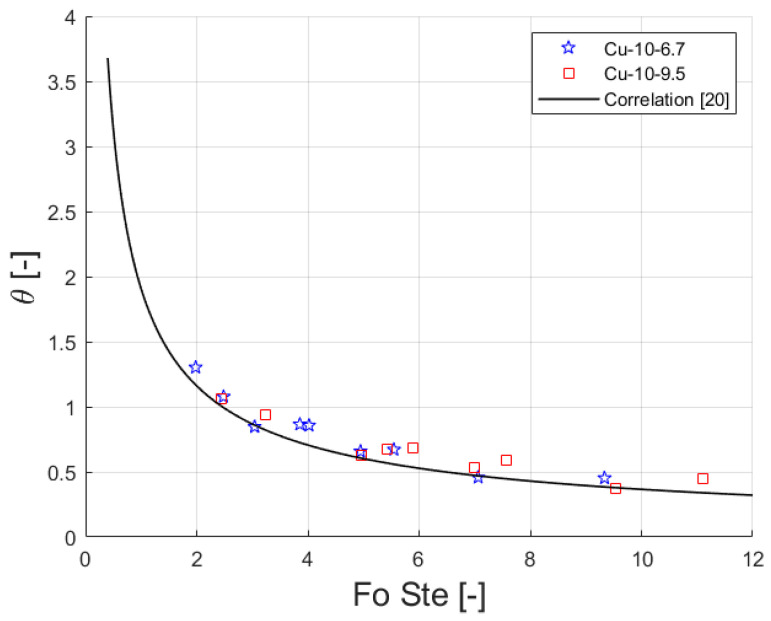
Dimensionless temperature versus product Fourier and Stefan numbers. Experimental values and predicted curve [20].

Figure 9 shows the dimensionless temperature plotted against the product between Fourier number and Stefan number for the experimental data as well as the trend of Equation (1). The correlation was developed from experimental data obtained during the melting of paraffins embedded in aluminum foams. The correlation is also able to predict the experimental values for copper foams in the sandwichlike arrangement. The correlation shows a relative, absolute and standard deviation of-11.2, 12.0 and 8.6%, respectively, for the actual data.

## 6. Conclusions

The paper investigated the melting of phase change materials (paraffins) embedded in two copper foams, having the same number of PPI (10) but different relative density. Three different melting temperatures were tested (42, 55 and 64 °C) and three different heat fluxes (10, 15 and 20 kW m^−2^) were supplied. Samples were laterally heated by means of an electrical heater.

The main findings are the following:

- The melting front is almost parallel to the heater, except in the last part of the test, where liquid paraffin tends to overcome the solid paraffin due to volume variation during phase change from solid to liquid conditions.
- The higher the heat flux, the sooner the PCM reaches a complete melting and the higher the temperature reached by the heated side.
- The higher the melting temperature, the longer the time needed to completely melt the PCM and the higher the temperature reached by the heated side. The optimum choice of the melting temperature of the PCM should consider the time during which the PCM/foam based heat sink will work.
- The range of tested relative densities is quite limited, and so there is a weak effect of this parameter on the actual experimental results. However, the trend seems to highlight that the higher the foam relative density, the better the thermal performance.
- The higher the thermal conductivity of the parent foam material, the lower the temperature reached by the heated side, and the parent foam material does not affect the time needed for a complete melt.
- The correlation proposed by Diani and Campanale [20] is also suitable for copper foams in a sandwichlike arrangement.

## Figures and Tables

**Table 1:** Geometrical parameters of the tested copper foams.

Parameter	Cu-10-6.7	Cu-10-9.5
Number of Pores Per Inch, PPI ^a^ (inch^−1^)	10	10
Relative Density, *ρ_r_* ^a^ (–)	0.067	0.095
Porosity, *ε* ^a^ (–)	0.933	0.905
Fiber Thickness, *t* ^b^ (mm)	0.390	0.403
Fiber Length, *l* ^b^ (mm)	1.583	1.378
Surface Area per Unit of Volume, *a_sv_* ^a^ (m^−1^)	698	831

^a^ Provided by the manufacturer [2]. ^b^ Measured by Mancin et al. [21].

**Table 2:** Thermophysical characteristics of the tested paraffins. Data by the manufacturer [22].

Property	RT42	RT55	RT64HC	
Melting Temperature Range	38–43	51–57	63–65	(°C)
Heat Storage Capacity ^a^	165	170	250	(kJ kg^−1^)
Specific Heat Capacity	2	2	2	(kJ kg^−1^ K^−1^)
Density Solid ^b^	0.88	0.88	0.88	(kg dm^−3^)
Density Liquid ^c^	0.76	0.77	0.78	(kg dm^−3^)
Thermal Conductivity (both phases)	0.2	0.2	0.2	(W m^−1^ K^−1^)
Volume Expansion	12.5	14	11	(%)

^a^ Combination of latent and sensible heat in a temperature range of 35 °C to 50 °C. ^b^ Evaluated at 15 °C for RT42 and RT55, at 20 °C for RT64HC. ^c^ Evaluated at 80 °C.

**Table 3:** Summary of the experimental results.

Experimental Test	*T_i_* (°C)	*T_f_* (°C)	*t_melt_* (s)
Cu-10-6.7, RT42, *HF* = 10 kW m^−2^	21.78	59.21	940
Cu-10-6.7, RT42, *HF* = 15 kW m^−2^	19.88	65.85	705
Cu-10-6.7, RT42, *HF* =20 kW m^−2^	19.68	71.07	558
Cu-10-6.7, RT55, *HF* = 10 kW m^−2^	20.67	70.71	1332
Cu-10-6.7, RT55, *HF* = 15 kW m^−2^	20.45	77.72	927
Cu-10-6.7, RT55, *HF* = 20 kW m^−2^	20.74	84.63	728
Cu-10-6.7, RT64HC, *HF* = 10 kW m^−2^	18.20	84.73	1941
Cu-10-6.7, RT64HC, *HF* = 15 kW m^−2^	21.25	92.71	1235
Cu-10-6.7, RT64HC, *HF* = 20 kW m^−2^	22.69	99.38	927
Cu-10-9.5, RT42, *HF* = 10 kW m^−2^	19.49	56.02	946
Cu-10-9.5, RT42, *HF* = 15 kW m^−2^	20.60	62.00	645
Cu-10-9.5, RT42, *HF* = 20 kW m^−2^	20.55	64.76	488
Cu-10-9.5, RT55, *HF* = 10 kW m^−2^	21.05	67.55	1245
Cu-10-9.5, RT55, *HF* = 15 kW m^−2^	19.65	73.70	876
Cu-10-9.5, RT55, *HF* = 20 kW m^−2^	19.75	78.91	682
Cu-10-9.5, RT64HC, *HF* = 10 kW m^−2^	22.66	82.68	1751
Cu-10-9.5, RT64HC, *HF* = 15 kW m^−2^	21.97	88.63	1172
Cu-10-6.7, RT64HC, *HF* = 20 kW m^−2^	21.36	93.19	899

## Data Availability

Data are contained within the article.
